# BMI and waist circumference cut-offs for corresponding levels of insulin sensitivity in a Middle Eastern immigrant versus a native Swedish population – the MEDIM population based study

**DOI:** 10.1186/s12889-016-3892-1

**Published:** 2016-12-09

**Authors:** Louise Bennet, Karin Stenkula, Samuel W Cushman, Kerstin Brismar

**Affiliations:** 1 Department of Clinical Sciences, Lund University, Lund, Sweden; 2 Department of Family Medicine, Lund University Skåne University Hospital, Lund, Sweden; 3 Glucose Transport and Protein Trafficking, Lund University, Lund, Sweden; 4 National Institute of Diabetes and Digestive and Kidney Diseases National Institutes of Health, Bethesda, MD USA; 5 Department of Molecular Medicine and Surgery, Karolinska Institutet, Stockholm, Sweden; 6 Karolinska University Hospital, Stockholm, Sweden; 7 Center for Primary Health Care Research Clinical Research Center, Skåne University Hospital, Malmö, Sweden

**Keywords:** Insulin sensitivity, Immigrants, Body mass index, Abdominal obesity

## Abstract

**Background:**

The aim of this study was to identify corresponding body mass index (BMI) and waist circumference cut-offs for equivalent levels of insulin sensitivity in a Middle Eastern immigrant population compared with native Swedes.

**Methods:**

Citizens of Malmö, Sweden aged 30 to 75 years, who were born in Iraq or Sweden, were in 2010–2012 invited to participate in a health examination including anthropometrics, oral glucose tolerance test, fasting samples and interviews concerning sociodemographic factors and lifestyle behaviours.

**Results:**

In total, 1176 individuals born in Iraq and 688 born in Sweden, without previously diagnosed type 2 diabetes, participated in the study. In normal weight participants (BMI < 25 kg/m^2^), 21.2% of Iraqis vs 9.3% of Swedes were insulin resistant. Corresponding figures in participants without abdominal obesity (waist circumference, men < 94 cm, women < 80 cm) were 28.2% of Iraqis vs 9.4% of Swedes.

The age-adjusted insulin sensitivity index (ISI) for obese Swedes (BMI 30 kg/m^2^) corresponded in Iraqi men with BMI of 28.5 kg/m^2^, and in Iraqi women with BMI of 27.5 kg/m^2^. The ISI level in abdominally obese Swedes corresponded with waist circumference cut-offs of 84.0 cm and 71.0 cm in Iraqi men and women, respectively.

In men only, larger waist circumference (*P*_interaction_ = 0.026) presented a stronger association with impaired ISI in Iraqis as compared to Swedes.

**Conclusions:**

Our data shows that the impact of BMI and waist circumference on ISI is ethnic- and gender-specific, indicating a disturbed fat metabolism in Iraqi males in particular. Our data suggests that 10 cm lower cut-off values for abdominal obesity, than is currently recommended by major organisations, should be considered when estimating diabetes risk in Middle Eastern populations.

**Electronic supplementary material:**

The online version of this article (doi:10.1186/s12889-016-3892-1) contains supplementary material, which is available to authorized users.

## Background

Type 2 diabetes prevalence is on the increase worldwide. In 2013 more than 380 million people were estimated to be suffering from the disease [1]. The most rapidly growing diabetes incidence is taking place in Asia, where it is estimated that it will have increased by 150% within the next few decades and consequently pose an enormous pressure on health economy and equity. This situation demands a heightened effort to understand the mechanisms driving type 2 diabetes development among different ethnic populations [1].

The Middle Eastern population constitutes the largest and fastest growing non-European immigrant group in Sweden today [2], with the vast majority residing in the cities of Stockholm and Malmö. Migration is a strong risk factor for cardiometabolic diseases [3, 4]. The recent MEDIM study (the impact of Migration and Ethnicity on Diabetes In Malmö) identified immigrants born in Iraq as a high risk population for type 2 diabetes [5]. Insulin sensitivity, together with insulin secretion, are the main determinants of type 2 diabetes [6], but the MEDIM study previously reported that insulin sensitivity (assessed as insulin sensitivity index, ISI) rather than insulin secretion, contributes to the high diabetes risk in the Iraqi immigrant population. Thus, ISI is a main target in the prevention of type 2 diabetes in this population [7]. Dysfunctional insulin sensitivity, or insulin resistance, is dependent on ectopic fat accumulation in liver, visceral adipose tissue, and skeletal muscle [8]. Although the Iraqi immigrant population is generally more obese (BMI > 30 kg/m^2^) than the native Swedish population (37.5% vs 23.0%, *p* < 0.001) [9], the reduced insulin sensitivity in this population is not fully explained by BMI or other cardiometabolic risk factors [7]. These findings are consistent with other studies reporting different fat distributions across ethnicities and consequently highlight the limitation of BMI as a measure of adiposity [10].

Identifying BMI and waist circumference cut-offs in Iraqi immigrants that equate to corresponding ISI levels in Swedes with obesity (BMI ≥ 30 kg/m^2^) or abdominal obesity (waist circumference, men ≥ 94 cm, women ≥ 80 cm) could provide a sharper tool in the clinical setting for identifying Middle Eastern men and women at increased risk for insulin resistance and type 2 diabetes. Our aims here were to study the ethnic- and gender-specific prevalence of insulin resistance in relation to BMI and waist-circumference, and further to establish ethnic- and gender-specific BMI and waist-circumference cut-offs for corresponding insulin sensitivity.

## Methods

### Subjects

**Fig. 1:**
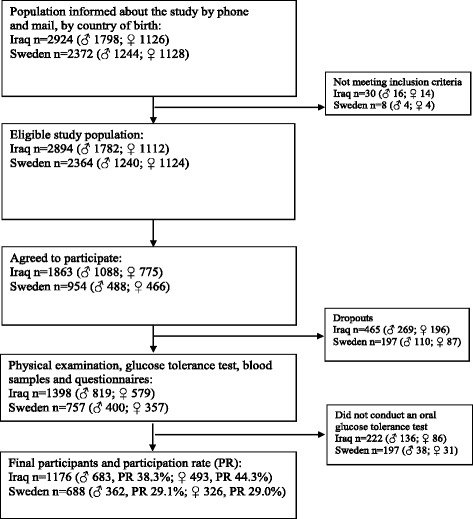
Flow diagram of the recruitment of the study population. PR, participation rate

The presented data was collected within the population-based MEDIM study conducted from 2010 to 2012 in the city of Malmö in southern Sweden. Malmö is a multicultural city with a third of its population born abroad, with the majority being born in Iraq [2]. As previously reported, citizens of Malmö, which were born in Iraq (Middle Eastern ethnicity) or born in Sweden (Caucasian ethnicity), aged 30 to 75 years were randomly selected from the census register and invited by post and phone to participate in the MEDIM population-based study [7]. Our aim was to recruit Iraqi-born and Swedish-born groups, matched for gender and age distribution. Individuals with previously known diabetes confirmed by medication with oral hypoglycaemic agents and/or insulin, or by a fasting glucose level of ≥7.0 mmol/L, were considered as having diabetes and excluded from the study. Furthermore, individuals with severe physical or mental illness or disabilities were also excluded. Examinations were conducted within a relatively short time-frame to minimise cohort effects and assessment biases (February 1, 2010 to December 31, 2012). A flow chart describing the recruitment process and participation rate is presented in Fig. 1.

### Materials and methods

#### Physical examination

Trained Swedish- and Arabic-speaking research nurses conducted standard physical examinations. Assessments of standard physical examinations and clinical variables such as blood pressure, height, weight, waist circumference, and BMI were performed as described previously [11].

#### Blood samples and oral glucose tolerance test

Participants were instructed not to eat or drink anything but water and not to use tobacco after 10 pm the day before testing; they were also asked to bring a record of their current medications to the examination. The following morning, a 75-g oral glucose tolerance test (OGTT) was performed. Blood samples were collected prior to glucose loading and at 30, 60, 90 and 120 min thereafter; glucose was measured in fresh plasma from venous whole blood immediately after sampling using a photometer (HemoCue AB, Ängelholm, Sweden) as described previously [11]. Plasma insulin, cholesterol, triglyceride (p-TG), high-density lipoprotein (p-HDL) and low-density-lipoprotein (p-LDL), C-peptide and high-sensitive c-reactive protein were determined as previously described [7, 11].

Mean glucose levels and mean insulin levels were calculated from fasting, at 30 min., 60 min. and 120 min. during the glucose load. Insulin sensitivity index (ISI), corrected insulin response (CIR) and oral disposition index (DIo) were assessed using the Matsuda indices calculated from the OGTT results as described previously [7]. ISI provides an estimate of insulin action, CIR provides an estimate of beta cell function and DIo provides an estimate of beta-cell function adjusted for insulin resistance, and is calculated as CIR multiplied by ISI [12].

### Questionnaires

Information on lifestyle habits (alcohol consumption, tobacco smoking, physical activity), sociodemographic factors (education level and economic difficulties), previous diagnosis of diabetes, current medication, family history of diabetes (in biological parents and/or siblings) was collected through interviews by Arabic- and Swedish-speaking nurses using structured questionnaires in Swedish and Arabic, as described previously in detail [7, 11].

### Definitions

‘Weight class’: BMI < 25 kg/m^2^ was considered as ‘normal weight’, BMI ≥ 25 kg/m^2^ and <30 kg/m^2^ as ‘overweight’ and BMI ≥ 30 kg/m^2^ as ‘obesity’ (11). The category ‘underweight’ was not applied in this study since only three out of 1176 born in Iraq and five out of 688 Swedes had BMI less than 18.5 kg/m^2^.

‘Abdominal obesity’ was considered in accordance with the World Health Organization (WHO) and International Diabetes Federation (IDF) [13, 14] criteria, which are based on an increased risk of metabolic complications in European and Middle Eastern populations at waist circumference ≥ 94 cm in men and ≥ 80 cm in women.

The ISI in the Swedish-born group was categorised into tertiles, i.e. three equally large groups; first tertile ISI <77.7 (‘insulin resistant’); second tertile ISI ≥ 77.7 or < 133.0 (‘intermediate’) and third tertile ISI ≥ 133.0 (‘insulin sensitive’). The Iraqi born participants were then, depending on their ISI levels, categorised into the corresponding groups.

### Questionnaires

Information on lifestyle habits, previous diagnosis of diabetes, current medication, family history of diabetes (in biological parents and/or siblings) and sociodemography was collected in interviews by Arabic- and Swedish-speaking nurses using structured questionnaires in Swedish and Arabic. All questionnaires were translated and back-translated by two independent professional translators with Arabic as their native language [11].

First degree family history of diabetes, smoking habits, alcohol consumption, hours physically active per week, economic difficulties and education level were categorised as described previously [7].

### Statistical analysis

Analyses were performed using STATA IC/12.1. Skewed variables were log_10_-transformed before analysis to approximate normal distributions. Differences in means were examined by linear regression analysis adjusting for age, whereas differences in proportions were adjusted for age using logistic regression.

ISI was log_10_ transformed due to the skewed distribution. Associations with ISI were assessed using multivariate linear regression analysis; data was expressed as beta coefficients (β) with 95% confidence intervals (CIs). Regression coefficients (β) for the continuous independent variables were standardised to a unit variance (per 1 standard deviation, SD), in strata of ethnicity and gender.

In Figs. 3 and 4, ISI was age adjusted and due to the skewed distribution log_10_ transformed. All tests were two-sided and a *p*-value of <0.05 was considered statistically significant. In order to minimise the multiple testing burden, interactions were considered only when the included marginal effects were significant. Multicollinearity was assessed using the variance inflation factor (VIF) but was not considered an issue as VIF values in the final multivariate regression models were <3.5.

## Results

### Gender-specific comparisons in cardio metabolic characteristics across ethnicity

In total, 1176 individuals born in Iraq (58.1% men) and 688 born in Sweden (52.6% men), not previously diagnosed with diabetes and of which all conducted an OGTT, were included in the study. Iraqi-born males and females presented with worse body fat measures assessed as BMI, waist circumference, waist-hip ratio, and waist-height ratio, as compared to their Swedish counterparts, Table 1. Obesity and abdominal obesity were most prevalent in Iraqi females, with 37.1 and 87.8% affected, respectively. Although Iraqi females presented with the worst body fat measures, Iraqi males had the highest triglyceride, mean glucose, C-peptide, serum insulin levels and further displayed the least beneficial insulin action and secretion, assessed as ISI, CIR and DIo, Table 1.

**Table 1:** Comparisons in cardiometabolic characteristics in women and men born in Iraq and Sweden participating in the MEDIM study 2010 to 2012

Variable	Swedish women	Iraqi women	*P*	Swedish men	Iraqi men	*P*
	*N* = 326	*N* = 493		*N* = 362	*N* = 683	
Age (years)	49.7 (11.1)	44.3 (8.9)	<0.001	48.9 (11.1)	46.0 (9.5)	<0.001
Body mass index, (kg/m^2^)	26.8 (5.1)	29.4 (4.8)	<0.001	27.2 (3.9)	28.8 (4.0)	<0.001
Waist circumference (cm)	88.4 (13.7)	92.0 (10.4)	0.001	97.1 (11.2)	98.4 (10.3)	0.034
Height (cm)	165.9 (7.0)	158.7 (6.1)	<0.001	179.2 (6.8)	172.8 (6.5)	<0.001
Waist to hip ratio	0.84 (0.1)	0.86 (0.06)	<0.001	0.93 (0.1)	0.94 (0.05)	0.270
Waist to height ratio	0.54 (0.08)	0.58 (0.06)	<0.001	0.54 (0.06)	0.57 (0.06)	<0.001
Obesity (BMI ≥ 30 kg/m^2^)	71 (21.8)	183 (37.1)	<0.001	77 (21.3)	228 (33.4)	<0.001
Abdominal obesity (waist men ≥ 94 cm; women ≥ 80 cm)	234 (71.8)	433 (87.8)	<0.001	222 (61.3)	460 (67.3)	0.064
Fasting glucose (mmol/L)	5.5 (0.6)	5.6 (0.7)	0.037	5.7 (1.1)	5.6 (0.8)	0.131
Mean glucose 0, 30, 60, 120 min (mmol/L)	6.8 (1.4)	7.0 (1.4)	0.013	7.0 (1.9)	7.3 (1.9)	0.005
Glucose 120 min (mmol/L)	5.9 (1.8)	6.1 (1.7)	0.108	5.6 (1.8)	5.7 (2.1)	0.406
Fasting insulin (mIE/L)	8.0 (5.0)	10.6 (6.9)	<0.001	10.5 (8.5)	12.15 (7.6)	0.001
Mean insulin 0, 30, 60, 120 min (mIE/L)	38.1 (23.9)	50.8 (32.6)	<0.001	47.5 (38.7)	62.6 (39.7)	<0.001
Serum insulin 120 min (mIE/L)	38.8 (30.6)	52.4 (49.3)	<0.001	49.8 (68.4)	61.5 (61.3)	0.005
C-peptide (nmol/l)	0.7 (0.3)	0.8 (0.3)	<0.001	0.7 (0.4)	0.9 (0.3)	0.001
Total cholesterol (mmol/L)^c^	5.3 (1.0)	4.8 (0.9)	<0.001	5.3 (1.1)	5.1 (0.9)	<0.001
p-LDL (mmol/L)^c^	3.3 (0.8)	3.1 (0.8)	0.001	3.4 (1.0)	3.4 (0.8)	0.305
p-HDL (mmol/L)^c^	1.6 (0.4)	1.3 (0.4)	<0.001	1.3 (0.4)	1.1 (0.3)	<0.001
p-TG (mmol/L)^c^	1.1 (0.5)	1.3 (0.7)	<0.001	1.4 (1.0)	1.7 (1.1)	<0.001
Insulin Sensitivity Index^a^	111.4 (80.6–168.9)	90.5 (57.2–131.2)	<0.001	90.1 (58.4–145. 2)	70.1 (47.3–104.0)	<0.001
Disposition Index ^a,b^	15232.4 (8910.1–26758.1)	13277.1 (7436.3–24268.0)	0.111	13495.8 (7741.0–22764.5))	12324.9 (6721.3–22514.4)	0.114
First-degree family history of diabetes, *n* (%)	91 (29.8)	263 (53.5)	0.001	87 (24.3)	325 (47.2)	0.001
Hours physically active/week	4.1 (2.5)	1.7 (2.0)	<0.001	4.1 (2.4)	2.0 (2.2)	<0.001
Smokers, *n* (%)	79 (25.9)	57 (11.6)	<0.001	91 (25.4)	227 (33.0)	0.010
Alcohol consumers, *n* (%)	232 (76.1)	24 (4.9)	<0.001	309 (86.3)	190 (27.6)	<0.001
Education level ≤ HS, *n* (%)	53 (17.4)	161 (32.7)	<0.001	70 (19.6)	158 (23.0)	0.173
Economic difficulties ≥ once in the last 12 months, *n* (%)	50 (16.4)	227 (46.1)	<0.001	53 (14.8)	377 (54.8)	<0.001
Time since migration, years		16 (11–21)	−		17 (12–24)	−

Data is presented in means (standard deviation, SD), numbers (percentages) or for non-normally distributed data medians (interquartile range, IQR). Differences in means between groups were adjusted for age using linear regression models (for continuous variables) while differences in proportions between groups (but for male gender and family history of diabetes) were studied using logistic regression adjusting for age.

All tests were two-sided and a *p*-value of <0.05 was considered statistically significant

^a^Data presented as IQR

^b^Disposition only included cases where the glucose level at 30 min was >4.44 mmol/l and greater than the fasting glucose level [33]

^c^Differences adjusted for treatment with medication lowering cholesterol levels (i.e. statins or similar medication)

Abbreviations: *Econ* diff. Economic difficulties, *DI* disposition index, *HDL* high density lipoprotein, *Hs*-*CRP* high sensitive C-reactive protein, *HS* high school, *IQR* interquartile range, *ISI* insulin sensitivity index, *LDL* low density lipoprotein, *SD* standard deviation, *TG* triglycerides

### Prevalence of insulin resistance in relation to abdominal obesity and weight class across ethnicity and gender

**Fig. 2:**
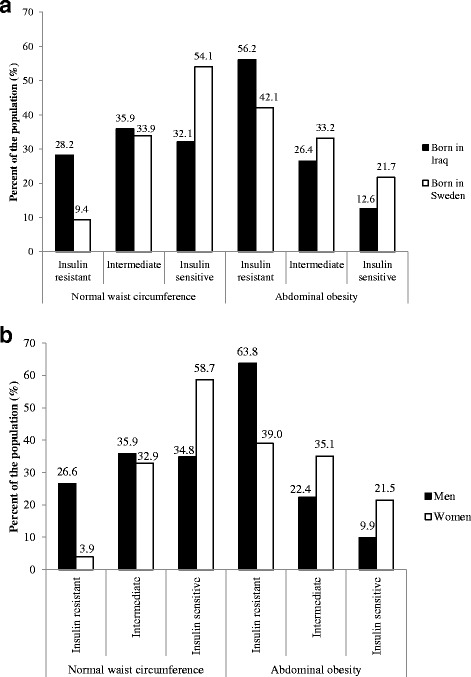
Ethnic- and gender-specific distribution of tertiles of Insulin Sensitivity Index (insulin resistant; intermediate; insulin sensitive) in relation to normal waist circumference and abdominal obesity across ethnicities (Panel **a**) and gender (Panel **b**) in participant of the MEDIM study

Abdominally obese Iraqi participants (Fig. 2a) and abdominally obese males (Fig. 2b) presented with the highest prevalence of insulin resistance (56.2 and 63.8%, respectively). In Iraqi participants, with normal waist circumference, the proportion of ‘insulin-resistant’, ‘intermediate’, and ‘insulin-sensitive’ individuals was evenly distributed as compared to Swedish participants, with almost 30% of normal-waist Iraqis being insulin resistant as compared to less than 10% of Swedes (Fig. 2a). This pattern remained when comparing males with females, showing a considerably higher proportion of males with normal-waist circumference being insulin resistant as compared to females (26.6 and 3.9%, respectively) Fig. 2b.

Categorising the participants into weight classes of ‘normal weight’, ‘overweight’ and ‘obesity’ we observed that insulin resistance was most prevalent in obese subjects, whether Iraqis (68%) or Swedes (67%). However, in ‘normal-‘or ‘overweight’ subjects a higher proportion of Iraqis, as compared to Swedes, were insulin resistant, but most apparent in normal weight Iraqis vs Swedes with twice as high prevalence of insulin resistance (normal weight Iraqis 21.2 vs Swedes 9.3%; overweight Iraqis 45.2 vs Swedes 31.8%). Irrespective of weight class, a larger proportion of males were insulin resistant as compared to females (normal weight males 19.8 vs females 8.9%; overweight males 48.9 vs females 28.8%; obese males 77.2 vs. females 57.2%.

### Gender-specific BMI and waist-circumference cut-offs for corresponding levels of insulin sensitivity across ethnicity

**Fig. 3:**
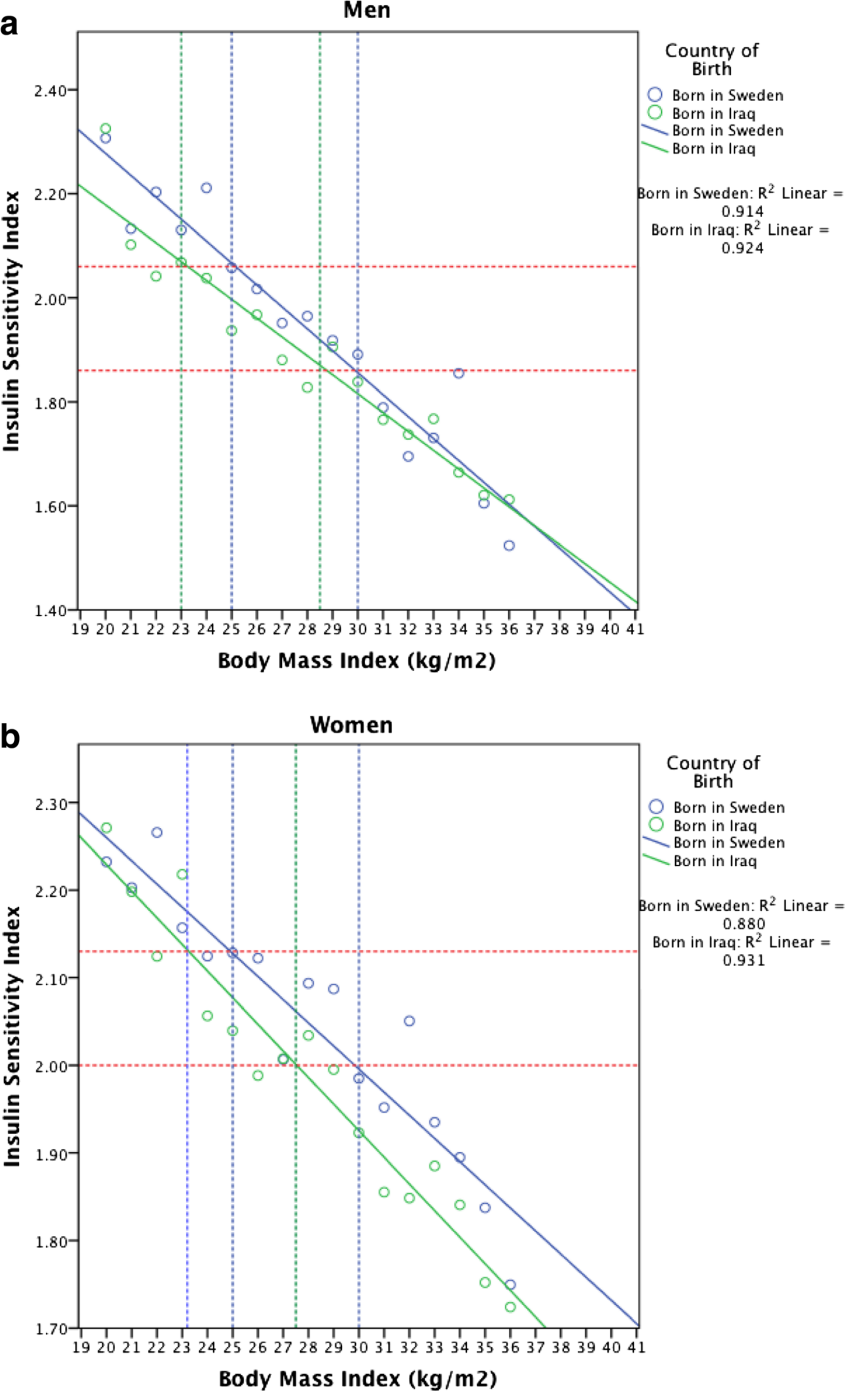
BMI cut-offs across ethnicities for corresponding levels of Insulin Sensitivity Index (age adjusted, log_10_ transformed) in overweight (BMI ≥ 25 kg/m^2^) and obese (BMI ≥ 30 kg/m^2^) men (Panel **a**) and women (Panel **b**)

The equivalent level of ISI observed in obese Swedes (at BMI 30 kg/m^2^), was observed in Iraqi men at BMI 28.5 kg/m^2^, and in Iraqi women at BMI 27.5 kg/m^2^. The equivalent level of ISI in overweight native Swedes (at BMI of 25 kg/m^2^) was observed at BMI 23.0 kg/m^2^ in both Iraqi men and women, Fig. 3.

**Fig. 4:**
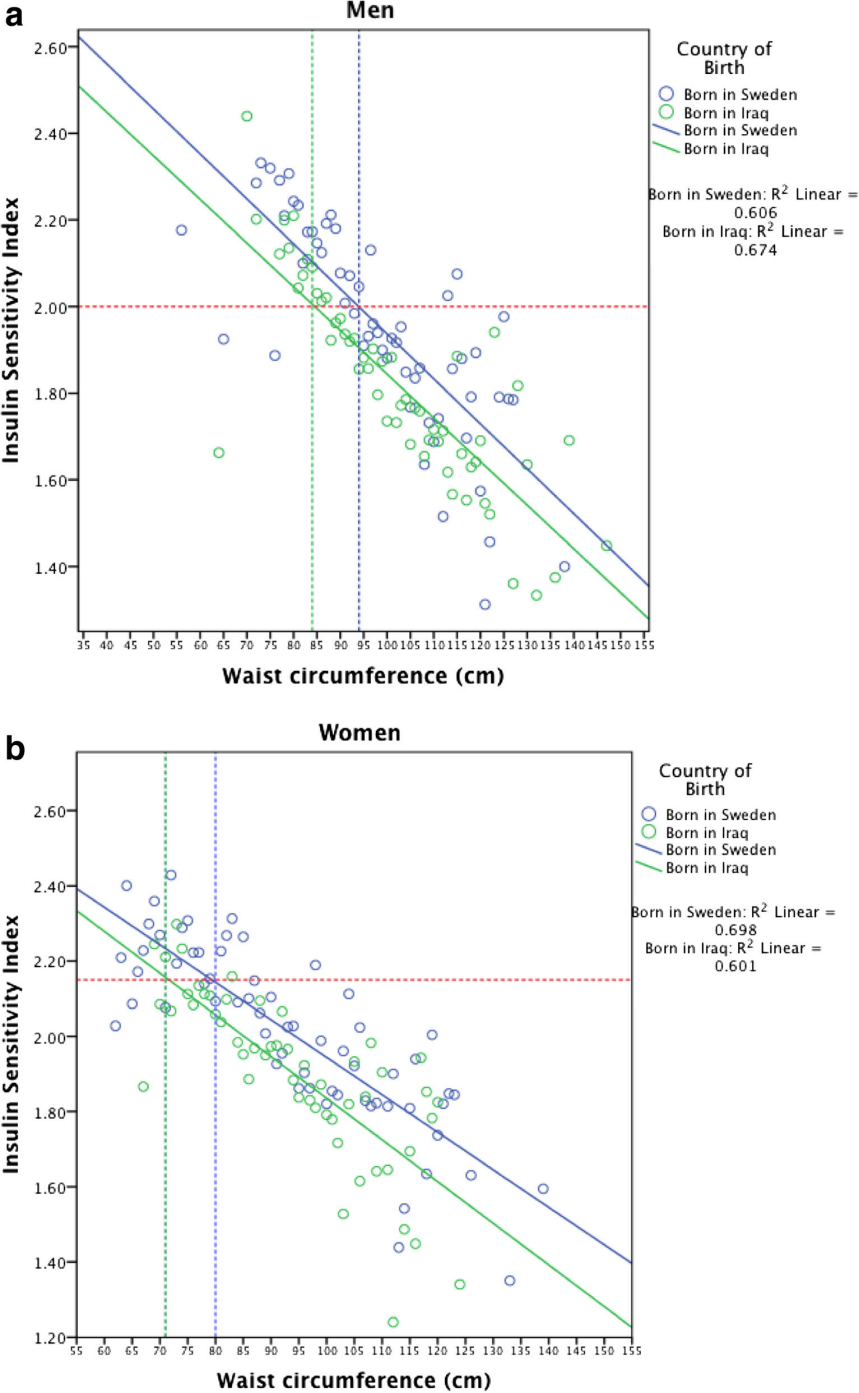
Waist circumference cut-offs across ethnicities for corresponding levels of Insulin Sensitivity Index (age adjusted, log10 transformed) in abdominally obese (men waist circumference ≥94 cm; women ≥80 cm) men (Panel **a**) and women (Panel **b**)

The equivalent levels of ISI seen at abdominal obesity in native Swedes (waist circumference, men ≥ 94 cm, women ≥ 80 cm) were observed at waist circumferences of 84.0 cm and 71.0 cm in Iraqi men and women, respectively (Fig. 4).

The equivalent level of DI in obese and overweight Swedes, corresponded with approximately four units lower BMI levels in Iraqis (Additional file 1: Figure S5). The equivalent levels of DI in abdominally obese native Swedes corresponded with waist circumference of 80 cm and 73 cm in Iraqi men and women respectively (Additional file 1: Figure S6).

### Associations between insulin sensitivity and type 2 diabetes related risk factors across ethnicity and gender

Iraqi ethnicity, male gender, waist circumference, plasma triglycerides, smoking, physical activity, and plasma HDL all represented diabetes-related risk factors independently associated with ISI (Additional file 1). Further, we identified a gender-specific interaction between male gender and plasma triglycerides (*P*_interaction_ = 0.042). In correspondence the association between ISI and plasma triglycerides was in a model stratified by gender found to be weaker in males than in females (men, β -0.039; women, β-0.052), Table 2.

**Table 2:** Association between insulin sensitivity index (ISI) and type 2 diabetes related risk factors in Iraqi and Swedish born participants in the MEDIM study 2010 to 2012

Risk factors	Total male study population			Interaction with Iraqi ancestry	Men born in Sweden			Men born in Iraq^1^		
	*N* = 840 (*R* ^2^ = 0.37)				*N* = 293 (*R* ^2^ = 0.46)			*N* = 547 (*R* ^2^ = 0.28)		
	β	95% CI		*P*_interaction_	β	95% CI		β	95% CI	
Born in Sweden	Reference									
Born in Iraq	−.102^***^	−.133	−.071	−	−			−		
Body mass index (kg/m^2^), per 1 SD	−.053^***^	−.082	−.025	0.001	−.108^***^	−.153	−.062	NS		
Waist circumference (cm), per 1 SD	−.077^***^	−.106	−.049	0.026	−.059^**^	−.104	−.015	−.112^***^	−.132	−.093
Plasma triglycerides (mmol/L), per 1 SD	−.039^***^	−0.54	−.024	0.042	−.056^***^	−.082	−.030	−.031^***^	−.050	−.012
Plasma HDL (mmol/L), per 1 SD	.021^**^	.005	0.37	NS	NS			.022^**^	.002	.042
Physical activity (hours/week), per 1 SD	.034^***^	.019	.049	NS	.045^***^	.021	.069	.027^**^	.008	.045
Current tobacco smoking								NS		
− No	Reference				Reference					
− Yes	.034^**^	.002	.066	NS	NS					
First-degree Family History of Diabetes										
− No	Reference							Reference		
− Yes	−.036^***^	−.067	−0.006	NS	NS			−.045^**^	−.082	−.009
Risk factors	Total female study population			Interaction with Iraqi ancestry	Females born in Sweden			Females born in Iraq^1^		
	*N* = 628 (*R* ^2^ = 0.39)				*N* = 245 (*R* ^2^ = 0.41)			*N* = 383 (*R* = 0.32)		
Born in Sweden	Reference									
Born in Iraq	−.141^***^	−.174	−.107	−	−			−		
Body mass index (kg/m^2^), per 1 SD	−.030^*^	−.060	−.001	NS	NS			−.039^*^	−.078	−.001
Waist circumference (cm), per 1 SD	−.090^***^	−.120	−.059	NS	−.126^***^	−.155	−.096	−.085^***^	−.123	−.047
Plasma triglycerides (mmol/L), per 1 SD	−.052^***^	−0.72	−.033	NS	−.061^***^	−.092	−.030	−.061^***^	−.078	−.001
Plasma HDL (mmol/L), per 1 SD	.022^**^	.004	.040	NS	NS			NS		
Physical activity (hours/week), per 1 SD	.018^*^	.001	.034	NS	NS			.027^**^	.005	.050

Data assessed by multivariate linear regression presented as the last step in backward elimination, entry and removal of covariates at *p* ≤ 0.05 and *p* ≤ 0.1. Models adjusted for being born in Iraq (the non-stratified total models), age, male gender (non-stratified models), body mass index, waist circumference, plasma triglycerides, plasma high density lipoprotein, high sensitive C-reactive protein, physical activity, current tobacco smoking, family history of diabetes in first degree relatives, economic difficulties. ^1^ For Iraqi born time since migration was also included as a covariate in the analysis

Regression coefficients (β) for the continuous independent variables, were standardised to a unit variance (per 1 standard deviation (SD) in the strata of ethnicity and gender

Interactions with country of birth were considered only when the included marginal effects were significant

Variance inflation factor (VIF) <1.5

^***^ *p* ≤ 0.001; ^**^ *p* ≤ 0.010; ^*^ *p* ≤ 0.050

Data presented for the total study population and for males and females separately

Data assessed by multivariate linear regression displaying β coefficients with 95% confidence intervals with ISI as the dependent variable

In males, the association between ISI and plasma triglycerides (*P*_interaction_ = 0.001), and between ISI and BMI (*P*_interaction_ = 0.001), was ethnic-dependent and weaker in those born in Iraq as compared to those born in Sweden (Table 2). Conversely, the association between ISI and waist circumference was stronger in males born in Iraq as compared to those born in Sweden, confirmed by an ethnic-specific interaction between Iraqi ethnicity and waist circumference (*P*_interaction_ = 0.026). Replacing waist circumference with waist-height-ratio, rather than waist-hip-ratio, gave consistent results in all models, with an interaction between waist-height-ratio and country of birth (*P*_interaction_ =0.016).

We found no significant interactions in females.

## Discussion

### Statement of principal findings

A novelty of the study is the estimation of BMI and waist circumference cut-offs based on corresponding levels of insulin resistance in a Middle Eastern versus Caucasian born population at-risk for diabetes. A key finding is that a third of normal weight and/or normal waist Iraqi immigrants are insulin resistant and that males are the strongest risk group. Another key finding is that waist-circumference cut-offs in Iraqis are approximately 10 cm below that of native Swedes, for the corresponding level of insulin sensitivity. We also report that the impact of BMI, waist-circumference and plasma triglycerides on insulin resistance differs across ethnicity and gender, indicating a disturbed fat metabolism in the Middle Eastern population in general, and in Middle Eastern men in particular, contributing to the profound insulin resistance in this immigrant population.

### Possible mechanisms for altered insulin resistance across ethnicities

Impaired insulin sensitivity is associated with fat accumulation in the visceral adipose tissue, liver, and skeletal muscle [8]. Waist-circumference or waist-height ratios are surrogate measures of visceral fat depot and our finding of a stronger association of waist circumference (or waist-height ratio) with insulin sensitivity in Iraqi-born, as compared to Swedish-born men, could be due to a more pronounced visceral fat depot and/or hepatic fat depot in the immigrant population. Iraqi participants also had higher plasma triglyceride levels than Swedish born participants, with the highest levels in Iraqi males. Further, our finding that plasma triglycerides displayed a stronger association with insulin sensitivity in males than in females, and in the immigrant population compared with native Swedes, indicates a disturbed fat metabolism in Iraqis in general, but in Iraqi males in particular.

Previous studies have implied different mechanisms involved in fat and glucose metabolism in populations of different ethnic backgrounds, such as differences across ethnicities in levels of adiponectin, leptin and fibroblast-growth factor (FGF) [15] playing mediating roles in the relationship between adiposity and insulin resistance [16], and subsequent progression to hyperglycaemia and type 2 diabetes [17, 18]. An ethnic- and gender-specific disturbed fat metabolism could explain our finding that Iraqi participants and males, without signs of overweight or abdominal obesity according to the definitions in Europeans [14], had twice the prevalence of insulin resistance as compared to their female counterparts.

Furthermore, very few participants born in Iraq with obesity or abdominal obesity (<5%) were insulin sensitive, indicating a low eligible proportion of participants with metabolic healthy obesity (MHO). There has been some debate on whether MHO really is as harmless for CVD risk as it has been proposed [19, 20]. An Iranian follow up study has also shown increased CVD risk in those with MHO [21], which highlights the possibility of ethnic differences in the impact of MHO on cardiometabolic disease, but this requires further study.

Still, even though it has not been addressed here, more knowledge of mechanisms regulating hepatic-, visceral-, and peripheral insulin resistance, and of biomarkers reflecting insulin resistance and metabolic profile, is needed in order to shed more light on mechanisms contributing to diabetes risk across ethnicities.

Although lower BMI and waist circumference levels should be considered, we conclude that both BMI and waist circumference/waist-height ratios may be too blunt an anthropometric measure to capture insulin resistance in this population. Others have shown that the sagittal abdominal diameter (SAD) is the most superior anthropometric measure predicting insulin resistance in Caucasian men [15], but future studies need to be conducted to identify the most superior anthropometric measurement for identifying Middle Eastern populations at high risk of insulin resistance.

### Body fat cut-offs and diabetes risk across ethnicities

Insulin secretion and action are the main determinants of type 2 diabetes [22], although with varying quantitative contributions in different ethnic populations. For instance, blacks are reported to be more insulin resistant compared to Caucasians, whereas South Asians are reported to be more insulin sensitive, but have poorer insulin secretion as compared to Caucasians, indicating differing mechanisms contributing to diabetes among ethnicities [23]. Diabetes risk in non-whites is equivalent at a lower waist circumference and/or BMI than for whites [24]. Several studies have consequently concluded that the cut-offs previously recommended by the WHO should be reduced when applied to non-Caucasian populations [25–30]. Our study contributes novel findings in that it compares Middle Eastern and Caucasian populations that have not yet developed diabetes, but are at high risk of doing so by being insulin resistant. The findings in this study show that the waist-circumference cut offs in Middle Eastern males and females are a decimetre below that of the recommended cut-offs in the prevention of the metabolic syndrome [14]. Our data are consistent with data from black African populations showing obesity-equivalent cut-offs of BMI 26 kg/m^2^ compared to Caucasians (BMI 30 kg/m^2^), but less consistent with diabetes risk cut-offs for South Asians (BMI of 22 kg/m^2^) [24]. Our findings thus indicates that Middle Eastern populations share metabolic characteristics with black, rather than with South Asian, populations. This is supported by previous findings that diabetes is strongly driven by insulin resistance in black (African) [23] and Middle Eastern populations [7] in contrast to South Asians, where diabetes is mostly driven by impaired insulin secretion rather than insulin resistance [23].

Although insulin secretion is higher in Iraqis than native Swedes, the MEDIM study has previously shown that it is not high enough to compensate for the profound insulin resistance in the Iraqi immigrant population as reflected by lower levels of disposition index in the normoglycaemic and prediabetic stages [7]. We have concluded that the lower disposition index in the non-diabetic immigrant population can contribute to their higher HbA1c levels and earlier diabetes onset [9]. Here we show that BMI and waist circumference levels for the corresponding levels of disposition index are even lower than for the corresponding levels of insulin resistance reflecting that we do not overestimate the impact of BMI and waist circumference on diabetes risk in the Iraqi born population.

### Strengths and limitations

A strength of our study is that the study populations of Iraqis and Swedes were recruited from the same neighbourhood, with the same access to tax subsidised health care, thus reducing the influence of a potentially socioeconomic bias. The participation rate was higher in the Iraqi immigrant population vs native Swedish population (approximately 40% vs. 30%). We have previously reported that the study sample is representative of the Iraqi immigrant population showing no differences in prevalence of type 2 diabetes between participants and non-participants [5]. The study sample is not only representative for the Iraqi immigrant population but also for the Iraqi population living in Iraq showing corresponding levels of type 2 diabetes [31]. We were also able to adjust for the influence of several confounding factors, although the influence of residual confounding could not be fully accounted for.

This study is unique in that it examines BMI and waist circumference cut-offs comparing a Middle Eastern with a Caucasian population, which has not been done previously. The study also assesses insulin resistance rather than type 2 diabetes, which provides a direct measurement of glycaemic control in relation to BMI and waist circumference in a population at risk for type 2 diabetes. A limitation is the cross sectional design making it impossible to draw conclusions regarding causality. Thus, the impact of waist circumference and BMI on diabetes risk should be tested prospectively. Another weakness of our study is that SAD, as proposed by previous studies as the most superior anthropometric measurement of insulin resistance, was not measured [15]. Furthermore, we have not studied body fat but surrogate anthropometric markers.

## Conclusions

Our data shows that a large proportion of non-obese Iraqi males and females are insulin resistant which implies a high risk for type 2 diabetes. The data presented herein supports a disturbed fat metabolism in Iraqis in general, and in Iraqi males in particular and highlights that at least 10 cm lower cut-off values for abdominal obesity than now recommended by major organisations should be considered when estimating diabetes risk in Middle Eastern populations.

## Additional files

Additional file 1: BMI and waist circumference cut offs for the corresponding level of Disposition Index across ethnicities. **Table**. Association between insulin sensitivity index (ISI) and type 2 diabetes related risk factors in the total study population participating in the MEDIM study 2010 to 2012. Data assessed by multivariate linear regression displaying β coefficients with 95% confidence intervals with ISI as the dependent variable. **Figure S5**. BMI cut-offs across ethnicities for corresponding levels of Disposition Index (age adjusted, log10 transformed) in overweight (BMI>25 kg/m2) and obese (BMI>30 kg/m2) men (Panel a) and women (Panel b). **Figure S6**. Waist circumference cut-offs across ethnicities for corresponding levels of Disposition Index (age adjusted, log10 transformed) in abdominally obese (men waist circumference ≥94 cm; women ≥80 cm) men (Panel a) and women (Panel b). (DOCX 133 kb)

## Abbreviations

BMI: Body mass index
CI: Confidence interval
DI: Disposition index
HDL: High density Lipoprotein
HS: High school
ISI: Insulin sensitivity index
LDL: Low density Lipoprotein
MHO: Metabolically healthy obese
OGTT: Oral glucose tolerance test
PA: Physical activity
SAD: Sagittal abdominal diameter
SD: Standard deviation
TG: Triglycerides
