# Supplementary material for: BMI and waist circumference cut-offs for corresponding levels of insulin sensitivity in a Middle Eastern immigrant versus a native Swedish population – the MEDIM population based study
Source: BMC Public Health. 2016 Dec 9;16:1242. doi: 10.1186/s12889-016-3892-1 (PMC5148840; doi:10.1186/s12889-016-3892-1)

**Additional file1**. Association between insulin sensitivity index (ISI) and type 2 diabetes related risk factors in the total study population participating in the MEDIM study 2010 to 2012.

Data assessed by multivariate linear regression displaying β coefficients with 95% confidence intervals with ISI as the dependent variable.

| **Risk factors** | **Total study population**  **N=1469 (R^2^=0.40)** | | | **Interaction with**  **male gender** |
| --- | --- | --- | --- | --- |
|  | **β** | **95% CI** | | ***P*_interaction_** |
| Born in Sweden  Born in Iraq | Reference  -.121^***^ | -.144 | -.099 | NS |
| Male Gender | -.096^***^ | -.118 | -.074 | - |
| Body mass index (kg/m^2^), per 1 SD | -.041^***^ | -.062 | -.021 | NS |
| Waist circumference (cm), per 1 SD | -.085^***^ | -.106 | -.065 | NS |
| Plasma triglycerides (mmol/L), per 1 SD | -.044^***^ | -0.56 | -.032 | 0.042 |
| Plasma HDL (mmol/L), per 1 SD | .024^**^ | .011 | .036 | NS |
| Physical activity (hours/week), per 1 SD | .027^***^ | .016 | .038 | NS |
| Current tobacco smoking |  |  |  |  |
| - No | Reference |  |  | Reference |
| - Yes | .036^**^ | .010 | .062 | NS |

**Additional file 1: Figure S5**. BMI cut-offs across ethnicities for corresponding levels of Disposition Index (age adjusted, log_10_ transformed) in overweight (BMI>25 kg/m^2^) and obese (BMI>30 kg/m^2^) men (Panel a) and women (Panel b).

**Panel a**


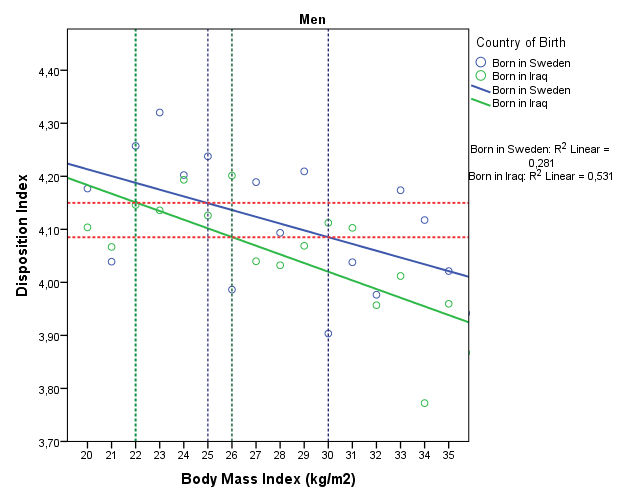


**Panel b**


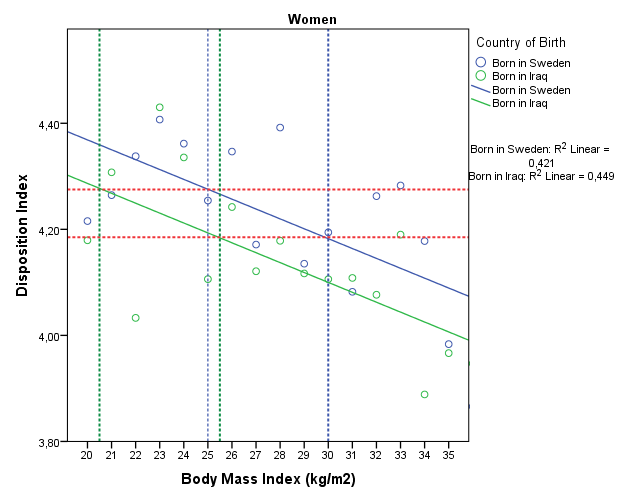


**Figure S6**. Waist circumference cut-offs across ethnicities for corresponding levels of Disposition Index (age adjusted, log10 transformed) in abdominally obese (men waist circumference ≥94 cm; women ≥80 cm) men (Panel a) and women (Panel b).

**Panel a**


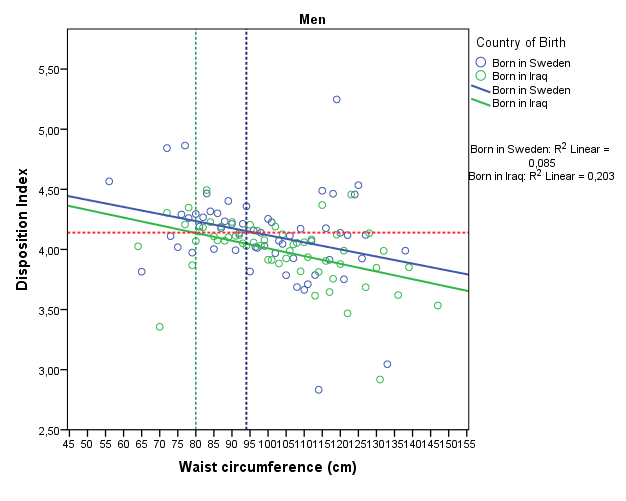


**Panel b**


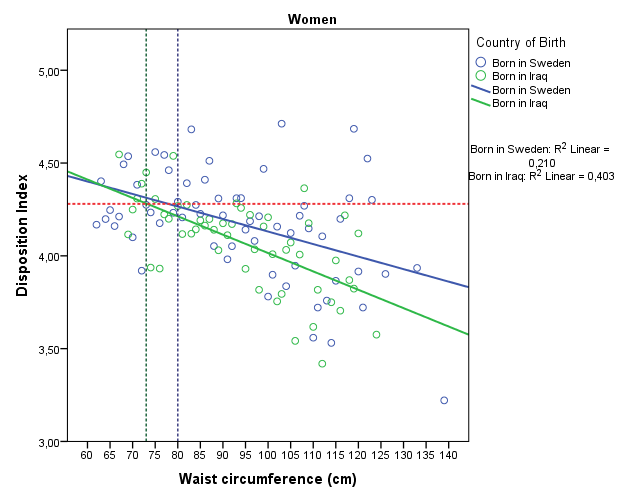

Supplement: Additional file 1: — BMI and waist circumference cut offs for the corresponding level of Disposition Index across ethnicities. Table. Association between insulin sensitivity index (ISI) and type 2 diabetes related risk factors in the total study population participating in the MEDIM study 2010 to 2012. Data assessed by multivariate linear regression displaying β coefficients with 95% confidence intervals with ISI as the dependent variable. Figure S5. BMI cut-offs across ethnicities for corresponding levels of Disposition Index (age adjusted, log10 transformed) in overweight (BMI>25 kg/m2) and obese (BMI>30 kg/m2) men (Panel a) and women (Panel b). Figure S6. Waist circumference cut-offs across ethnicities for corresponding levels of Disposition Index (age adjusted, log10 transformed) in abdominally obese (men waist circumference ≥94 cm; women ≥80 cm) men (Panel a) and women (Panel b). (DOCX 133 kb) [file 12889_2016_3892_MOESM1_ESM.docx]
